# Genome wide association study to detect genetic regions related to isoflavone content in a mutant soybean population derived from radiation breeding

**DOI:** 10.3389/fpls.2022.968466

**Published:** 2022-08-18

**Authors:** Jung Min Kim, Jae Il Lyu, Dong-Gun Kim, Nguyen Ngoc Hung, Ji Su Seo, Joon-Woo Ahn, You Jin Lim, Seok Hyun Eom, Bo-Keun Ha, Soon-Jae Kwon

**Affiliations:** ^1^Advanced Radiation Technology Institute, Korea Atomic Energy Research Institute, Jeongeup, South Korea; ^2^Division of Plant Biotechnology, Chonnam National University, Gwangju, South Korea; ^3^Department of Horticulture, College of Industrial Sciences, Kongju National University, Yesan, South Korea; ^4^Department of Horticultural Biotechnology, Institute of Life Sciences & Resources, Kyung Hee University, Yongin, South Korea

**Keywords:** Isoflavone, SNPs, QTLs, GBS, GWAS, MDP

## Abstract

Isoflavones are major secondary metabolites that are exclusively produced by legumes, including soybean. Soy isoflavones play important roles in human health as well as in the plant defense system. The isoflavone content is influenced by minor-effect quantitative trait loci, which interact with polygenetic and environmental factors. It has been difficult to clarify the regulation of isoflavone biosynthesis because of its complex heritability and the influence of external factors. Here, using a genotype-by-sequencing-based genome-wide association mapping study, 189 mutant soybean genotypes (the mutant diversity pool, MDP) were genotyped on the basis of 25,646 high-quality single nucleotide polymorphisms (SNPs) with minor allele frequency of >0.01 except for missing data. All the accessions were phenotyped by determining the contents of 12 isoflavones in the soybean seeds in two consecutive years (2020 and 2021). Then, quantitative trait nucleotides (QTNs) related to isoflavone contents were identified and validated using multi-locus GWAS models. A total of 112 and 46 QTNs related to isoflavone contents were detected by multiple MLM-based models in 2020 and 2021, respectively. Of these, 12 and 5 QTNs were related to more than two types of isoflavones in 2020 and 2021, respectively. Forty-four QTNs were detected within the 441-Kb physical interval surrounding Gm05:38940662. Of them, four QTNs (Gm05:38936166, Gm05:38936167, Gm05:38940662, and Gm05:38940717) were located at Glyma.05g206900 and Glyma.05g207000, which encode glutathione S-transferase THETA 1 (*GmGSTT1*), as determined from previous quantitative trait loci annotations and the literature. We detected substantial differences in the transcript levels of *GmGSTT1* and two other core genes (*IFS1* and *IFS2*) in the isoflavone biosynthetic pathway between the original cultivar and its mutant. The results of this study provide new information about the factors affecting isoflavone contents in soybean seeds and will be useful for breeding soybean lines with high and stable concentrations of isoflavones.

## Introduction

Soybean [*Glycine max* (L.) Merr] is a major source of protein and oil as well as secondary metabolites that are beneficial for human health (Liang et al., 2010). Soy isoflavones primarily consist of daidzein, glycitein, genistein, daidzin, glycitin, genistin, 6-o-malonyldaidzin, 6-o-malonylglycitin, 6-o-malonylgenistin, 6-o-acetyldaidzin, 6-o-acetylglycitin, and 6-o-acetylgenistin in seeds, and coumestrol in leaves and roots (Kudou et al., 1991; Yuk et al., 2011). Soy isoflavones are synthesized and conjugated through a branch of the phenylpropanoid pathway, and are stored in the central vacuole of cells (Dastmalchi and Dhaubhadel, 2014). Phytochemicals are metabolites that are naturally derived from plants. Some of them, such as phenolic compounds and anthocyanins, have antioxidant and antiviral properties (Messina, 1999; Lee et al., 2005). Soy isoflavones have diverse functions. They interact with rhizobia to promote nodulation in the roots, root nodule symbiosis for nitrogen fixation, and are involved in resistance to pathogens and stresses (Graham et al., 1990; Sreevidya et al., 2006; Li et al., 2013). Recent studies have shown that consumption of products and medicines derived from soybean is beneficial for human health (Kang et al., 2002; Collison, 2008; Lobato et al., 2012). Soy isoflavones, as a class of phytoestrogens, have antitumor activity and the ability to ameliorate the symptoms of cardiovascular disease and climacteric syndrome, as well as relieving post-menopausal symptoms (Farina et al., 2006; Ma et al., 2008).

The detailed mechanism of the regulation of isoflavone biosynthesis is still unclear because complex quantitative traits such as isoflavone content, tocopherol content, and saponin content are interrelated, and are affected by genotype, year, and location (Rupasinghe et al., 2003; Gutierrez-Gonzalez et al., 2009; Shaw et al., 2016). Both additive and epistatic effects are affected by genotype × environment interactions, and because many alleles make small contributions to overall phenotypic variations, it is difficult to identify quantitative trait loci (QTLs) related to isoflavone content (Gutierrez-Gonzalez et al., 2011; Fan et al., 2015). To address these problems, high-density genotyping platforms and advanced next-generation sequencing (NGS) technologies have been developed and applied to detect genetic polymorphisms related to traits of interest in many crops (Thomson, 2014; Rasheed et al., 2017). Genotype-by-sequencing (GBS) can be applied in situations where there are multiple samples with high genetic diversity and large genomes. Compared with whole genome genotyping, GBS is more cost-effective, simpler, highly reproducible, and can partially capture important parts of the genome that are otherwise inaccessible. This approach has been used to discover huge numbers of single nucleotide polymorphisms (SNPs). It is anticipated that high-density SNP genotyping will be compatible with genome-wide association study (GWAS), marker-assisted selection, and genomic selection for crop improvement (Elshire et al., 2011; He et al., 2014).

To map QTLs associated with isoflavone content, previous studies conducted QTL mapping using an F_2_ population and recombinant inbred lines (RILs) as a bi-parental population. Primomo et al. (2005) identified QTLs and epistatic interactions underlying isoflavone contents in soybean seed using RILs with 99 polymorphic simple sequence repeat (SSR) markers and detected 17 QTLs spanning multiple loci. Cai et al. (2018) detected 48 additive QTLs underlying variations in seed isoflavone content using SNP and SSR markers. Using specific-locus amplified fragment sequencing (SLAF-seq) methods, Pei et al. (2018) constructed a high-density genetic map using 3,541 SLAF markers and data from multiple environments and detected 24 stable QTLs for isoflavone components. Although linkage mapping has revealed some core QTLs underlying qualitative and quantitative traits such as agronomic traits (Brummer et al., 1997; Chen et al., 2007), stress tolerance (Concibido et al., 2004; Lee et al., 2004), and some functional components (Warrington et al., 2015; Li et al., 2017), the low crossover rates of the two parents’ alleles in the F_2_ and RIL populations leads to limited segregation and recombination frequency, resulting in genetic distortion, low allelic diversity, and low mapping resolution (Zhang et al., 2010; Xu et al., 2017).

As an alternative to linkage mapping, GWAS has some advantages as a tool because of its high-density resolution, its suitability for analyses of large sample and population sizes, and the large amount of markers. In addition, it involves powerful statistical methods that analyze the association mapping (AM) between traits of interests and the amount of SNPs markers based on natural populations and the structure of linkage disequilibrium (LD) (Korte and Farlow, 2013; Ibrahim et al., 2020). In previous AM studies on soy isoflavones, Chu et al. (2017) detected a gene encoding an R2R3-type MYB transcription factor (*GmMYB29*) that affected isoflavone content *via* GWAS and 207,608 SNPs using the NJAU 355K SoySNP array. This transcription factor binds to the promoters of two major genes in the isoflavone biosynthetic pathway, *IFS2* and *CHS8*, and activates their expression. Meng et al. (2016) detected 44 QTLs on chromosome 16 related to seed isoflavone content using restricted two-stage multi-locus GWAS with 55,340 SNPs derived from restriction-site-association DNA sequencing. Recently, Wu et al. (2020) identified 87 SNPs and 37 QTLs underlying isoflavone content using both GWAS and linkage analysis based on 28,926 SNPs and 4412 bins, respectively, and one major locus (qISO8-1) of *GmMPK1* was significantly co-localized on chromosome 8.

The spontaneous mutation rate is as low as one mutation per 100 million to 1 billion nucleotides per generation. Ionizing radiation can increase the mutation rate by up to 1,000-fold to 1 million-fold compared with the natural mutation rate (Johnson et al., 2000; Jiang and Ramachandran, 2010). Therefore, mutation breeding has been used by breeders and researchers to rapidly generate elite cultivars and high-quality, large mutant populations. This method has been used to genetically improve many crops. To date, approximately 3,365 varieties of more than 210 species have been developed using mutation technology (Malek et al., 2014; Chaudhary et al., 2019).^1^ Using gamma-ray radiation, Kim et al. (2019) developed a high-stearic acid mutant soybean Hfa180, with a stearic acid content more than 8.57-fold that in the original cultivar. Joshi et al. (2016) selected two mutant rice lines, D100-200 and D100-209, with increased salt tolerance; they showed increases of 18 and 34% in yield and related parameters compared with wild-type plants under saline conditions. Lee et al. (2014) generated a lipoxygenase-free soybean mutant H70 with reduced undesirable flavors. This mutant had a deletion and single-point mutations of *Lox1*, *Lox2*, and *Lox3* genes compared with the original cultivar. The soybean mutant population used in this study was developed as a mutant diversity pool (MDP) by our group. Its agronomic traits, contents of functional components, and expression levels of genes related to certain phytochemicals have been described in other studies (Kim et al., 2020, 2021).

No previous studies have conducted AM using multiple GWAS models and a mutant soybean population. In this study, the first aim was to dissect causative QTLs related to isoflavone content in soybean seeds using an MDP and multiple GWAS methods; MLM and mrMLM (mrMLM, FastmrMLM, FASTmrEMMA, pLAR-mEB, pKWmEB, and ISIS EM-BLASSO) under year-to-year variation. A total of 25,646 high-quality SNPs in 189 mutant soybean genotypes with phenotypic data were used for this GBS-based GWAS analysis to detect important SNPs. The results of this study provide comprehensive insights into the factors that affect isoflavone contents in soybean seeds.

## Materials and methods

### Mapping population

The association mapping population consisting of 189 soybean lines from the MDP was selected and successfully validated with target region amplification polymorphism (TRAP) markers. Briefly, for constructing a soybean MDP, 1000 M_1_ seeds of each accession were derived from seven cultivars by 250 Gy of gamma-irradiation (Co^60^) and planted at Radiation Breeding Research Farm in the Korea Atomic Energy Research Institute (KAERI, 35.5699°N 126.9722°E, Jeongeup, Jeollabuk, Republic of Korea). Subsequently, 1695 M_1:2_ seeds single-seed descent planted in soybean filed at the KAERI during M_1_–M_5_ generation and then we selected 523 accessions based on agronomic traits. To homozygous population, 523 M_5:6_ seeds were propagated as bulks until M_12_ generation to remove redundant phenotypes. Finally, we selected 208 soybean mutants showing genetically fixed morphological characteristics. Previous GWAS of this population have revealed details of its genetic diversity, important agronomic traits, and functional genes (Kim et al., 2020, 2021). The seeds of the MDP were obtained from the (KAERI). To evaluate year-to-year variations in isoflavone contents, field trials were conducted in 2020 and 2021 at the Radiation Breeding Research Farm at the KAERI. In each year, the 189-member MDP was planted with a randomized complete block design with three replications. Each genotype was sown in one row per plot, and each row was 300 cm long, with 80 cm spacing between rows. Field management during the growing season, including fertilization, weed management, and insecticide-fungicide application were based on a standard cropping system. After maturity, three individual plants were randomly harvested from each plot and the seeds were collected to quantify isoflavones.

### Analysis of isoflavone contents in soybean seeds

Isoflavones were extracted and quantified as described by Chen et al. (2020) and Lim et al. (2021), with slight modifications. Briefly, 10 dried seeds from each genotype were ground into a fine powder using a 2010 GENO/GRINDER (SPEX samplePrep LLC, Metuchen, NJ, United States). Then, 20 mg of the powder was placed in a 2-mL microcentrifuge tube with 1 mL of 80% (v/v) methanol. The mixture was incubated in a shaking incubator for 24 h (room temperature, 150 rpm). After centrifugation at 13,000 rpm for 5 min, the supernatant (1-mL) was passed through a syringe filter (NORM-JECT, Henke-Sass Wolf, Tuttlingen, Germany) equipped with a 0.22 μm PTFE membrane (GVS, Sanford, ME, United States). The filtered extract was placed in a 2-mL vial and subjected to ultra-performance liquid chromatography (UPLC) analysis. The UPLC was equipped with a reversed-phase UPLC column (ACQUITY BEH C18 Column; 2.1 × 50 mm, 1.7 μm particle size, Waters Corp., Milford, MA, United States). The mobile phase consisted of A (water with 0.1% v/v formic acid) and B (acetonitrile with 0.1% v/v formic acid). The elution gradient was as follows: 2 min of equilibration at 13%, followed by a linear increase in mobile phase B to 17% in 3 min, to 22% in 3 min, to 60% in 6 min, then held for 1 min (total run time, 20 min). The flow rate was 0.25 mL min^–1^, the injection volume was 1 μL, and the oven temperature was 25°C. Twelve isoflavone standards (daidzein (DZE), glycitein (GLE), Genistein (GNE), daidzin (DZI), glycitin (GLI), genistin (GNI), malonyl daidzin (MDZI), malonyl glycitin (MGLI), malonyl genistin (MGNI), acetyl daidzin (ADGI), acetyl glycitin (AGLI), and acetyl genistin (AGNI)) were purchased from Sigma-Aldrich for identification and quantification of each isoflavone in the soybean seeds.

### Statistical analysis of isoflavone contents

Descriptive statistics such as maximum, minimum, mean, standard deviation (SD), skewness and kurtosis were calculated for data from each of the two years using Microsoft EXCEL 2016. Two-way analysis of variance (two-way ANOVA) and Pearson’s correlation coefficients among individuals and total isoflavone contents with each year were estimated to determine the effects of genotype, year, and genotype × year using R with the following basic codes; aov (formula, data = NULL, projections = FALSE, qr = TRUE, contrasts = NULL) function and corr_coef (data, verbose = TRUE). These analyses were conducted using the METAN package. The broad-sense heritability (*h*^2^) was calculated as follows:

$$h^{2}=\sigma^{2}\,\text{G}/\left(\sigma^{2}\,\text{G}+\sigma^{2}\,\text{GE}/n+\sigma^{2}\,\text{e}/n\,r\right)$$

where σ^2^_G_ is genotypic variance, σ^2^_GE_ is the variance of the genotype × environment interaction, σ^2^_e_ is error variance, *n* is the number of environments, and r is the number of replications within each environment. The frequency distribution of phenotypic data was displayed using ggpubr and cowplot in the R package.

### Single nucleotide polymorphisms genotyping and genotype-by-sequencing-based genome-wide association study

The 189 members of the MDP were genotyped using 37,249 SNPs derived by GBS as described by Kim (2021). The raw GBS data have been deposited at the NCBI sequence Read Archive (SRA) database (accession no. PRJNA845013). For the GWAS, a total of 25,646 high-quality SNPs in the 189 MDP with minor allele frequency (MAF) > 0.01 were filtered from raw SNPs using Plink (command settings: geno 0.5, mind 0.2). First, when applying the mixed linear model (MLM) and multi-locus random-SNP-effect mixed linear model (mrMLM) to reduce the false-positive rate in GWAS, a principal component analysis (PCA) was conducted and a kinship matrix (K) was calculated using Tassel 5.2 software to estimate the population structure. The Bonferroni threshold (*p*-value = 1.95 × 10^–6^) was calculated by negative log_10_ (0.05/n, n is the number of SNPs) and its threshold was used to detect important quantitative trait nucleotides (QTNs). A MLM based on single-locus effects was applied by Tassel using phenotype and genotype data with PC as a fixed effect and K as a random effect. In this study, we used six approaches: mrMLM, FastmrMLM, FASTmrEMMA, pLAR-mEB, pKWmEB, and ISIS EM-BLASSO within the multi-locus random-SNP-effect mixed linear model (mrMLM).^2^ The logarithm of the odds (LOD) score was set to 3 for determining significant QTNs and all options were set to default values. Information about the position of QTNs was retrieved from the Phytozome v9.1^3^ and Soybase^4^ databases, based on the cultivar Williams 82.a2.v1. The Manhattan and QQ plots were drawn using CMplot in the R package.

### RNA extraction and cDNA synthesis

The immature soybean seeds of wild type (Danbaek) and its mutant (DB-088) were collected into conical tubes, immediately frozen in liquid nitrogen, and then finely ground using a mortar and pestle. Total RNA was isolated using TRIZOL reagent (Invitrogen, Carlsbad, CA, United States). To adjust the concentration in the next two steps (RNA DNase treatment and cDNA synthesis), RNA quantity and quality were determined using a NanoDrop ND-1000 spectrophotometer (Thermo Fisher Scientific, Waltham, MA, United States). First, 20 μg total RNA was digested to remove contaminating DNA, and then the first-strand cDNA was synthesized using SuperScript III First-Strand synthesis SuperMix (Invitrogen). All of these procedures were conducted according to the manufacturer’s instructions.

### Gene transcript analysis quantitative real-time PCR

The transcript levels of *GmGSTT1, IFS1*, and *IFS2* were determined using the Bio-Rad CFX96 Real-time PCR system (Bio-Rad, Hercules, CA, United States) with iTaq Universal SYBR Green Supermix (Bio-Rad). The reference gene was *ELF1B* (Jian et al., 2008; Le et al., 2012). The thermal cycling conditions were as follows: 95°C for 10 min, followed by 40 cycles of 95°C for 15 s, 50°C for 15 s, and 72°C for 30 s. Each reaction had five replicates. The relative transcript level of each gene was calculated as follows:

$$2^{-\Delta \,\Delta \,\mathrm{CT}}={\left(C_{T,T\,a\,r\,g\,e\,t}-C_{T,N\,o\,r\,m}\right)}_{s\,a\,m\,p\,l\,e}-{\left(C_{T,T\,a\,r\,g\,e\,t}-C_{T,N\,o\,r\,m}\right)}_{c\,a\,l\,i\,b\,r\,a\,t\,o\,r}\,\left(\text{Rao et al., 2013}\right).$$

The primer sets used in this study are listed in Supplementary Table 1.

## Results

### Quantification of isoflavone content in soybean seeds across 2 years

To evaluate and validate the range of variation in isoflavone contents among genotypes and across years, the 189 mutant soybean genotypes were grown for 2 consecutive years (2020 and 2021) for GWAS analysis. We quantified the total isoflavones (TI) content and the contents of twelve types of isoflavones in soybean seeds: aglycones (DZE, GLE, and GNE), glucosides (DZI, GLI, and GNI), malonyl-glucosides (MDZI, MGLI, and MGNI), and acetyl-glucosides (ADGI, AGLI, and AGNI).

The average contents of isoflavones in each year were as follows: DZE [2020 (6.14 μg g^–1^), 2021 (4.67 μg g^–1^)], GLE [2020 (14.66), 2021 (2.80)], GNE [2020 (7.85), 2021 (6.75)], DZI [2020 (309.14), 2021 (168.45)], GLI [2020 (128.96), 2021 (109.00)], GNI [2020 (421.80), 2021 (252.62)], MDZI [2020 (796.50), 2021 (602.93)], MGLI [2020 (229.07), 2021 (226.86)], MGNI [2020 (1070.55), 2021 (885.91)], ADZI [2020 (117.03), 2021 (88.59)], AGNI [2020 (3.08), 2021 (2.41)], and TI [2020 (3104.80), 2021 (2350.98)].

**FIGURE 1:**
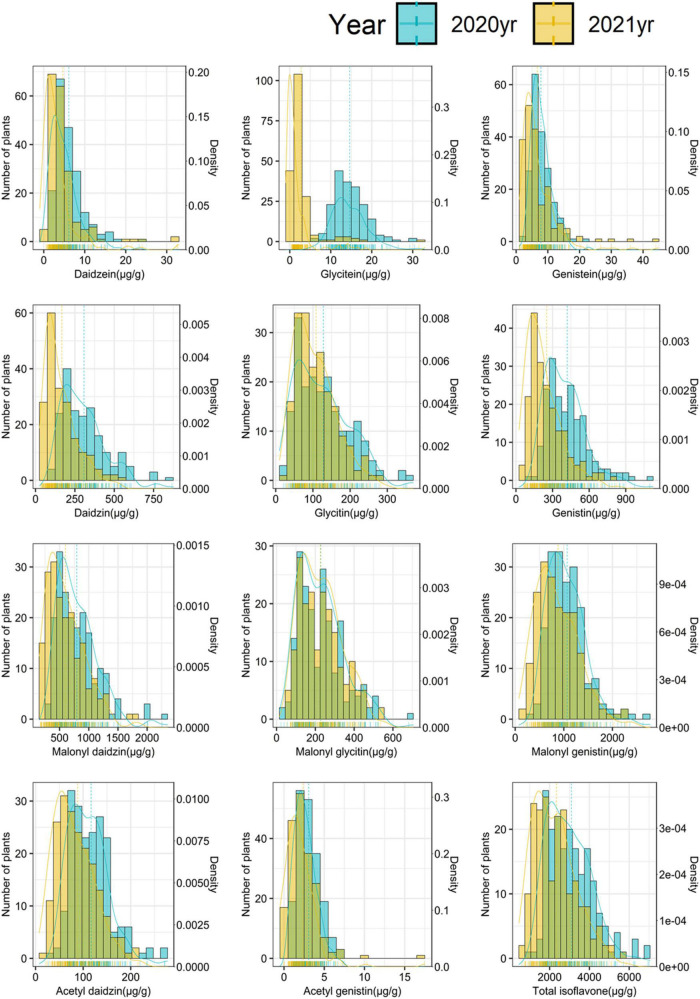
Frequency distribution of individual isoflavones and total isoflavone contents in mutant diversity pool (MDP).

Overall, isoflavone contents were lower in 2021 than in 2020. The average contents of aglycones, glucosides, malonyl-glucosides, acetyl-glucosides, and total isoflavones decreased from 9.55 μg g^–1^ in 2020 to 4.74 μg g^–1^ in 2021 (decreased by 50.39%), from 286.63 μg g^–1^ in 2020 to 176.69 μg g^–1^ in 2021 (decreased by 38.36%), from 698.71 μg g^–1^ in 2020 to 571.90 μg g^–1^ in 2021 (decreased by 18.15%), from 60.06 μg g^–1^ to 45.50 μg g^–1^ (decreased by 24.24%) and from 3104.80 μg g^–1^ in 2020 to 2350.98 μg g^–1^ in 2021 (decreased by 24.28%), respectively. The isoflavone contents showed continuous variations with normal distribution in the 2 years (Figure 1), indicating that isoflavone contents are quantitative traits that are regulated by numerous genes with smaller effects of environmental factors.

As shown in Table 1, the ANOVA revealed significant effects of genotype, year, and genotype × year on isoflavone contents. The contents of all isoflavones except for MGLI differed significantly between 2020 and 2021 (*P* < 0.001). This result suggested that the isoflavone content was strongly affected by genetic factors and environmental factors. Based on variance among genotypes, years, and genotype × year, the broad-sense heritability values calculated for isoflavone contents ranged from high (e.g., 86% for MGNI and for ADZI) to low (e.g., 15% for GLE). This indicated that heritability varied depending on the type of isoflavone, and that all of the studied isoflavones except for GLE and AGNI were more affected by genetic factors than by environmental factors.

**TABLE 1:** Descriptive statistics, ANOVA analysis, and broad-sense heritability of isoflavone contents in MDP.

Trait	Year	Min^a^	Max^a^	SD^a^	Mean^a^	Effects			Skewness	Kurtosis	*H* ^2^
						g^b^	y^c^	g × y^d^			
DZE	2020	1.33	23.26	3.40	6.14	***	***	***	1.71	4.28	0.51
	2021	0.48	32.66	4.47	4.67				3.73	18.05	
GLE	2020	5.61	30.40	4.00	14.66	***	***	***	0.86	1.56	0.15
	2021	0.33	31.53	3.66	2.80				4.28	23.77	
GNE	2020	2.62	22.35	3.15	7.85	***	***	***	1.15	1.92	0.47
	2021	1.00	44.04	5.61	6.75				3.19	14.91	
DZI	2020	88.15	866.08	142.15	309.14	***	***	***	1.11	1.37	0.81
	2021	33.82	525.19	102.75	168.45				1.25	1.28	
GLI	2020	20.83	352.50	68.98	128.96	***	***	***	0.76	0.19	0.78
	2021	28.86	282.53	51.71	109.00				0.87	0.41	
GNI	2020	119.38	1097.23	165.12	421.80	***	***	***	1.15	1.68	0.81
	2021	62.30	822.62	138.84	252.62				1.33	1.97	
MDZI	2020	264.50	2254.16	341.12	796.50	***	***	***	1.32	2.52	0.82
	2021	177.22	1757.39	302.93	602.93				1.02	1.12	
MGLI	2020	22.98	681.26	115.17	229.07	***	ns	***	0.90	0.72	0.77
	2021	52.15	535.40	101.86	226.86				0.61	–0.21	
MGNI	2020	256.15	2628.58	377.59	1070.55	***	***	***	1.09	1.91	0.85
	2021	172.84	2295.43	404.65	885.91				0.81	0.64	
ADZI	2020	26.89	271.71	40.56	117.03	***	***	***	0.96	1.59	0.86
	2021	20.68	217.18	39.14	88.59				0.76	0.31	
AGNI	2020	0.89	7.43	1.31	3.08	***	***	***	0.80	0.49	0.25
	2021	0	17.46	1.93	2.41				3.16	20.21	
TI	2020	1032.95	6993.06	1129.29	3104.80	***	***	***	0.90	0.81	0.86
	2021	617.35	5844.52	1036.56	2350.98				0.74	0.17	

DZE = daidzein; GLE, glycitein; GNE, Genistein; DZI, daidzin; GLI, glycitin; GNI, genistin; MDZI, malonyl daidzin; MGLI, malonyl glycitin; MGNI, malonyl genistin; ADGI, acetyl daidzin; AGLI, acetyl glycitin; AGNI, acetyl genistin; ns, not significant; H^2^, broad-sense heritability. *** significant at *p* < 0.001.

^a^ μg g^–1^.

^b^ g means the genotype effects.

^c^ y means the year effects.

^d^ g × y means the genotype by year effects.

### Correlation analysis between individual isoflavones in 2020 and 2021

**FIGURE 2:**
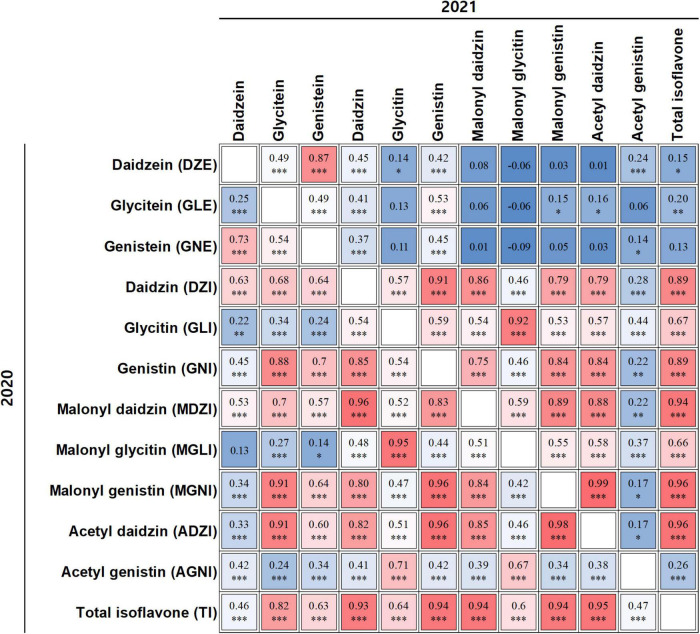
Correlation analyses among individual isoflavones and total isoflavones. Correlation coefficient in upper panel relates to isoflavone content data from 2021; that in lower panel relates to isoflavone content data from 2020. *, **, ***, and indicate significance at *p* < 0.05, 0.01, 0.001, and not significant, respectively.

To determine the relationships between total isoflavones and individual isoflavones, and between pairs of individual isoflavones in the two years, we conducted Pearson’s correlation analysis using data for 12 individual isoflavones (Figure 2). Strong positive correlations were detected between TI and ADZI [2020 (*r* = 0.95, *P* < 0.001) and 2021 (*r* = 0.96, *P* < 0.001)], MGNI [2020 (*r* = 0.94, *P* < 0.001) and 2021 (*r* = 0.96, *P* < 0.001)], MDZI [2020 (*r* = 0.94, *P* < 0.001) and 2021 (*r* = 0.94, *P* < 0.001)], GNI [2020 (*r* = 0.94, *P* < 0.001) and 2021 (*r* = 0.89, *P* < 0.001)], and DZI [2020 (*r* = 0.93, *P* < 0.001) and 2021 (*r* = 0.89, *P* < 0.001)]. This result indicated that the main contributors to TI were ADZI, MGNI, MDZI, GNI, and DZI in both years.

We also detected positive correlations between DZI and GNI, between DZI and MDZI (*r* = 0.85–0.96, *P* < 0.001) and between GLI and MGLI (*r* = 0.92–0.95, *P* < 0.001). GNI and MDZI were positively correlated with MGNI and ADZI (*r* = 0.84–0.96, *P* < 0.001). Although GLE and GNE were positively correlated with MGNI and ADZI (*r* = 0.60–0.91, *P* < 0.001), GLE and GNE were positively correlated with GNI (*r* = 0.7–0.88, *P* < 0.001), and DZI was positively correlated with others (*r* = 0.63–0.68, *P* < 0.001) in 2020, these correlations were not significant or weak in 2021. This result indicated that correlations among isoflavones were subject to year effects as well as genetic factors.

### Identification of quantitative trait nucleotides for total and individual isoflavone contents

**FIGURE 3:**
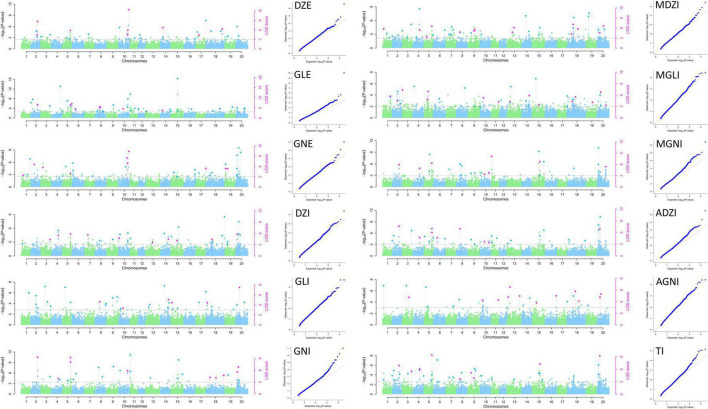
Manhattan and QQ plots for isoflavone contents in 2020 in the MDP using MLM and mrMLM.

**FIGURE 4:**
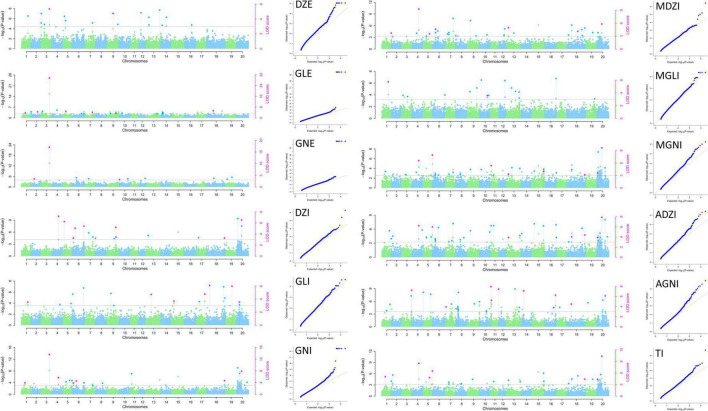
Manhattan and QQ plots for isoflavone contents in 2021 in the MDP using MLM and mrMLM.

The 25,646 SNPs (MAF < 0.01) were distributed across all the chromosomes, with frequencies ranging from 865 SNPs (3.37%, Chr. 12) to 2182 SNPs (8.51%, Chr. 18) per chromosome, with an average of 1,282 SNPs per chromosome. Thus, the SNPs were evenly distributed across all 20 soybean chromosomes in the MDP, and their number and distribution were sufficient for GWAS analysis. Of the 158 QTNs detected, 112 and 46 significant QTNs (LOD ≥ 3, *p*-value = 1.95 × 10^–6^) related to isoflavone contents were detected in 2020 and 2021, respectively. They were distributed across all chromosomes except for Chr. 12 and Chr. 17. The QTNs were detected using MLM and mrMLM methods (Figures 3, 4). Information for all QTNs is shown in Supplementary Tables 2, 3.

We detected 112 QTNs related to isoflavone contents in 2020. These QTNs were located on 18 of the 20 soybean chromosomes (all except Chr. 12 and 17). The number of QTNs per chromosome ranged from one (Chr. 1, Chr. 6, Chr. 7, and Chr. 16) to 19 (Chr. 5 and Chr. 20). Of the QTNs significantly related to isoflavones detected in 2020 (LOD ≥ 3, -log_10_(*P*) ≥ 5.7), nine QTNs were related to DZE and explained 1.71–27.89% of the variation in DZE content (–1.80 to 16.51 μg g^–1^). They were located on Chr. 2, 5, 10, 14, 18, and 20. The 10 QTNs for GLE were located on chromosomes 2, 5, 8, 9, 10, 11, and 20, and explained 1.67%–17.25% of the variation in GLE content (–3.05 to 8.93 μg g^–1^). Ten QTNs for GNE were located on Chr. 3, 5, 10, 19, and 20, and explained 2.52–22.62% of the variation in GNE content (–2.25 to 12.91 μg g^–1^). The nine QTNs for DZI were located on chromosomes 4, 5, 7, 14, 15, 18, and 20 and explained 6.50–18.18% of variation in QTN content (–117.49 to 385.37 μg g^–1^). The three QTNs for GLI were located on Chr. 14, 18, and 20 and explained 5.86–21.53% of the variation in QTN content (22.45–73.22 μg g^–1^). The 13 QTNs for GNI were located on chromosomes 2, 5, 10, 11, 15, 18, and 20 and explained 0.16–22.36% of the variation in GNI content (–324.75 to 525.77 μg g^–1^). The 12 QTNs for MDZI were located on Chr. 4, 5, 8, 9, 13, 18, and 20, and explained 2.07–16.67% of the variation in MDZI content (–316.48 to 891.52 μg g^–1^). The 8 QTNs for MGLI were located on chromosomes 1, 2, 3, 5, 14, 18, and 20 and explained 2.29–16.74% of the variation in MGLI content (–64.00 to 106.65 μg g^–1^). The 11 QTNs for MGNI were located on chromosomes 2, 4, 5, 11, 15, and 20, and explained 2.12–21.77% of the variation in MGNI content (–999.45 to 1261.72 μg g^–1^). The 12 QTNs for ADZI were located on chromosomes 2, 4, 5, 6, 8, 11, 15, and 20, and explained 3.80–19.31% of the variation in ADZI content (–31.76 to 77.35 μg g^–1^). The seven QTNs for AGNI were located on chromosomes 11, 13, 16, 18 and 20, and explained 4.21–14.11% of the variation in AGNI content (–0.60 to 0.57 μg g^–1^). The 8 QTNs for TI were located on chromosomes 2, 3, 4, 5, 9, 15, and 20, and explained 2.81–18.31% of the variation in TI content (–3115.80 to 2665.47 μg g^–1^).

Of the 122 QTNs detected in 2020, 12 QTNs were detected for two or more types of isoflavones and using more than one GWAS analysis method. These 12 QTNs were located on Chr. 2, 4, 5, 8, 9, 10, 11, 14, 15, and 20 (Table 2).

**TABLE 2:** Information about co-detected QTNs for isoflavone contents in 2020 using MLM-based models.

QTN	Trait	Chr	Position (bp)	Effect	-log10(P)	R2	MAF	Allele	Method	Candidate gene	Symbol	Description	References
Gm02:38740302	GLE	2	38740302	4.34 ∼6.41	6.25 ∼10.26	1.67 ∼17.25	0.50	C/A	1, 2, 4, 6	Glyma.02g202300		Pseudouridine synthase family protein	
	GNI			263.71 ∼307.91	5.78 ∼12.16	13.07 ∼22.36	0.02 ∼0.50		4, 6, 7				
	MGNI			465.57, 504.72	6.07, 7.48	2.12, 9.9	0.50		1, 4				
	ADZI			59.96	11.40	9.75	0.50		4				
	TI			1199.89 ∼1680.98	6.40 ∼9.15	2.82 ∼15.73	0.50		1, 2, 4, 6				
Gm04:26325280	DZI	4	26325280	–58.85	7.60	7.48	0.20	A/T	1	Glyma.04g144300	ENO1	Enolase 1	Arias et al., 2022
	MDZI			–179.27	12.73	5.74	0.20		1				
	TI			–428.11	6.96	3.64	0.20		1				
Gm04:30558742	DZI	4	30558742	351.75, 385.37	6.13 ∼7.23	10.04 ∼10.72	0.50	C/T	2, 4, 6				
	MDZI			750.06 ∼891.52	6.25, 10.9	8.02, 8.56	0.50		2, 4				
	MGNI			831.46, 843.02	5.78, 5.93	7.01, 9.87	0.50		4,6				
	ADZI			77.35	6.14	4.12	0.50		4				
	TI			2331.81 ∼2665.47	6.54 ∼8.49	6.04 ∼9.9	0.50		2, 4, 6				
Gm05:35170270	GNI	5	35170270	525.77	5.87	0.16	0.03	G/A	7	Glyma.05g160000	CESA8	Cellulose synthase family protein	Nawaz et al., 2017
	MGNI			1261.72	6.05	0.17	0.03		7				
Gm05:38940662	GNE	5	38940662	–0.95	5.79	5.74	0.50	T/C	2	Glyma.05g207000	GSTT1	Glutathione S-transferase THETA 1	Knizia et al., 2021
	DZI			–117.49 ∼50.67	6.22 ∼8.41	8.76 ∼14.28	0.50		2, 3, 4				
	GNI			–83.91 ∼64.22	9.82, 10.48	9.47, 13.47	0.50		1, 2				
	MDZI			–316.48 ∼85.11	6.32 ∼11.74	3.09 ∼10.23	0.50		1, 2, 3, 4, 5, 6				
	MGNI			–318.26 ∼125.76	6.33 ∼8.70	4.27 ∼14.98	0.50		1, 2, 3, 4, 6				
	ADZI			–10.93, 13.81	6.47, 9.32	11.54, 19.17	0.50		5, 6				
	TI			–837.84to ∼318.64	5.96 ∼12.95	6.22 ∼13.84	0.50		1, 2, 3, 4, 5				
Gm05:39039365	GLE	5	39039365	–3.05, 1.46	7.21, 10.00	6.26, 13.64	0.50	C/T	3, 4				
	GNI			–152.06to ∼47.36	5.84 ∼12.93	8.33 ∼19.88	0.50		3, 4, 5, 6				
	ADZI			–31.76 ∼13.62	5.80 ∼9.93	6.47 ∼14.38	0.50		1, 3, 4				
Gm05:41272024	GLE	5	41272024	–2.59	10.60	9.13	0.08	A/T	4				
	GNI			–324.75	5.77	17.04	0.08		7				
	MGNI			–831.48	5.75	17.47	0.08		7				
Gm08:12085018	MDZI	8	12085018	–115.85	5.70	4.85	0.36	T/G	2	Glyma.08g156300			
	ADZI			–12.79, 16.47	5.73, 9.94	4.65, 7.92	0.35, 0.36		1, 2				
Gm09:38479648	GLE	9	38479648	–1.7	7.25	10.71	0.16	T/C	2	Glyma.09g160600	RKL1	Receptor-like kinase 1	
	MDZI			–151.53	7.86	9.28	0.16		6				
Gm10:45467630	DZE	10	45467630	6.46, 8.69	5.01, 6.81	4.36, 8.21	0.50	A/C	4, 6	Glyma.10g223800		Basic-leucine zipper (bZIP) transcription factor family protein	Guo et al., 2021
	GLE			8.93	6.17	11.19	0.50		5				
	GNE			8.15 ∼9.52	7.54 ∼8.03	8.41 ∼9.52	0.50		1, 4, 6				
	GNI			397.33, 452.23	6.68, 7.82	5.82, 10.91	0.05		1, 2				
Gm10:49323094	DZE	10	49323094	12.44 ∼16.51	6.23 ∼21.67	16.85 ∼27.89	0.01 ∼0.50	C/T	1, 2, 4, 6, 7	Glyma.10g271000		Protein of unknown function (DUF1218)	
	GNE			9.88 ∼12.91	9.56 ∼12.28	12.97 ∼22.62	0.05		1, 2, 4, 6				
Gm11:508733	GNI	11	508733	–84.23	6.59	3.42	0.05	A/G	4	Glyma.11g007200			
	ADZI			–28.11	5.90	10.00	0.05		1				
Gm11:589619	GLE	11	589619	–2.67	12.71	14.88	0.07	T/A	2				
	GNI			–115.94	10.84	17.65	0.07		6				
	MGNI			–207.58, 250.58	7.47, 10.66	11.55, 11.61	0.07		4, 6				
Gm14:38439549	DZI	14	38439549	128.52, 147.19	6.08, 7.34	11.55, 13.48	0.50	A/G	4,6				
	GLI			73.22	6.56	20.44	0.50		2				
Gm15:24347624	GNI	15	24347624	–137.63	9.52	4.64	0.04	C/T	1	Glyma.15g200200	NHX2	Sodium hydrogen exchanger 2	
	TI			–745.38	10.76	5.32	0.04		4				
Gm15:24347662	MGNI	15	24347662	–206.83 ∼223.68	6.59 ∼8.43	6.76 ∼11.11	0.06	T/C	2, 4, 6	Glyma.15g200200	NHX2	Sodium hydrogen exchanger 2	
	ADZI			–21.53	5.95	9.23	0.06		6				
Gm15:47928380	GNI	15	47928380	170.09	5.78	8.60	0.13	T/C	3	Glyma.15g251200		CONTAINS InterPro DOMAIN/s: Retrotransposon gag protein	
	MGNI			287.69, 438.17	6.34, 7.57	0.20, 10.91	0.06, 0.13		2, 7				
	ADZI			23.82, 45.19	6.09, 7.44	0.19, 10.06	0.06, 0.13		3, 7				
Gm20:8188732	ADZI	20	8188732	37.23	7.54	9.66	0.17	G/C	3				
	TI			425.48	7.93	18.31	0.17		5				
Gm20:8460978	GNE	20	8460978	1.51	10.16	11.18	0.30	T/G	1				
	GNI			56.95	7.42	4.32	0.30		1				
	MGNI			304.24	6.83	8.27	0.30		3				
Gm20:8666294	DZE	20	8666294	0.97	7.96	3.72	0.20	G/A	4	Glyma.20g046800	ATCDPMEK	4-(cytidine 5’-phospho)-2-C-methyl-D-erithritol kinase	Jahan, 2019
	DZI			50.37,124.23	6.56, 6.95	8.99, 10.75	0.20		3, 5				
	MDZI			123.66	6.40	16.67	0.20		5				
	MGNI			168.65	11.37	21.77	0.20		5				
	ADZI			17.30	8.84	14.51	0.20		6				
Gm20:16191481	GNI	20	16191481	53.12, 68.88	7.24, 8.18	4.91, 13.55	0.20	A/G	4, 6				
	MDZI			122.93, 136.86	7.21, 13.50	7.46, 8.23	0.20		4, 6				
	ADZI			14.32 ∼15.48	7.78 ∼10.48	6.74 ∼10.27	0.20 ∼0.21		1, 2, 4				

DZE, daidzein; GLE, glycitein; GNE, Genistein; DZI, daidzin; GLI, glycitin; GNI, genistin; MDZI, malonyl daidzin; MGLI, malonyl glycitin; MGNI, malonyl genistin; ADGI, acetyl daidzin; AGLI, acetyl glycitin; AGNI, acetyl genistin; QTN, Quantitative trait nucleotide; Chr, chromosome; Effect, QTN effect for isoflavone content (μg g^–1^); R^2^, phenotype variation; MAF, minor allele frequency. Method 1–7 refers to mrMLM, FastmrMLM, FASTmrEMMA, pLAR-mEB, pKWmEB, and MLM, respectively.

We detected 46 QTNs related to isoflavones content in 2021. They were located on 16 of the 20 soybean chromosomes (all except Chr. 2, 8, 12, and 15). The one QTNs related to DZE were located on Chr. 3 and explained 12.30% of the variation in DZE content (16.30 μg g^–1^). The three QTNs for GLE were located on Chr. 3, 7 and 18 and explained 6.07–58.24% of the variation in GLE content (9.22–29.05 μg g^–1^). The two QTNs for GNE were located on Chr. 3 and 10, and explained 17.00–57.37% of the variation in GNE content (16.00–39.51 μg g^–1^). The six QTNs for DZI were located on Chr. 4, 5, 6, 9, and 20, and explained 2.95–19.67% of the variation in DZI content (–41.34 to 366.70 μg g^–1^). The three QTNs for GLI were located on Chr. 17, 18, and 19, and explained 5.74–25.26% of the variation in GLI content (–27.80 to 19.54 μg g^–1^). The five QTNs for GNI were located on Chr. 3, 4, 6, 18, and 20, and explained 4.66–26.02% of the variation in GNI content (–75.44 to 617.89 μg g^–1^). The four QTNs for MDZI were located on Chr. 4, 7, 13, and 20, and explained 6.04–22.13% of the variation in MDZI content (–116.13 to 1241.39 μg g^–1^). One QTN for MGLI was located on Chr. 1, and explained 4.01% of the variation in MGLI content (–140.64 μg g^–1^). The four QTNs for MGNI were located on Chr. 1, 4, 5, and 20, and explained 6.37–21.93% of the variation in MGNI content (–245.86 to 1199.61 μg g^–1^). The five QTNs for ADZI were located on Chr. 4, 5, 18, and 20, and explained 5.23–21.18% of the variation in ADZI content (–19.42 to 105.52 μg g^–1^). The seven QTNs for AGNI were located on Chr. 3, 11, 14, 16, and 20, and explained 8.13–34.48% of the variation in AGNI content (–1.60 to 3.31 μg g^–1^). The five QTNs for TI were located on Chr. 1, 4, 5, and 20 and explained 4.34–21.35% of the variation in TI content (–524.76 to 3212.93 μg g^–1^).

Of the 46 QTN detected in 2021, 5 QTNs were detected for two or more types of isoflavones and using more than one GWAS analysis method. These 5 QTNs were located on Chr. 3, 4, 5, 18, and 20 (Table 3).

**TABLE 3:** Information about co-detected QTNs for isoflavone contents in 2021 using MLM-based models.

QTN	Trait	Chr	Position (bp)	Effect	-log10(*P*)	R^2^	MAF	Allele	Method	Candidate gene	Symbol	Description	References
Gm03:40639077	DZE	3	40639077	16.30	7.12	12.30	0.50	C/A	1	Glyma.03g196800		Nucleic acid-binding, OB-fold-like protein	
	GLE			26.84 ∼29.05	11.33 ∼34.53	0.35 ∼58.24	0.01 ∼0.50		1, 2, 4, 7				
	GNE			33.69 ∼39.51	10.28 ∼20.15	31.69 ∼57.37	0.50		1, 2, 4, 7				
	GNI			613.86 ∼617.89	7.09 ∼15.78	23.00 ∼26.02	0.01 ∼0.50		1, 2, 4, 7				
Gm04:30558742	DZI	4	30558742	281.11 ∼366.70	6.36 ∼9.94	13.85 ∼19.36	0.50	C/T	1, 2, 4, 6				
	GNI			325.03 ∼374.79	6.50 ∼7.50	7.03 ∼12.12	0.50		2, 4, 6				
	MDZI			971.73 ∼1241.39	9.03 ∼10.26	14.44 ∼22.13	0.50		1, 2, 4, 6				
	MGNI			975.91 ∼1199.61	7.15 ∼7.96	7.38 ∼15.97	0.50		1, 2, 4, 6				
	ADZI			89.22 ∼105.52	6.97 ∼9.14	5.86 ∼9.46	0.50		1, 2, 4, 6				
	TI			2839.91 ∼3212.93	7.09 ∼10.72	11.79 ∼14.44	0.50		1, 2, 4, 6, 7				
Gm05:41272024	MGNI	5	41272024	–245.86 to ∼319.44	6.64 ∼10.23	9.43 ∼18.83	0.08	A/T	1, 2, 4, 6				
	ADZI			–19.42, 23.10	6.82, 9.50	5.69, 12.88	0.08		1, 6				
	TI			–524.76 to ∼603.99	6.81 ∼9.36	6.86 ∼13.42	0.08		2, 4, 6				
Gm18:56278301	GNI	18	56278301	–46.47, ∼38.91	5.88, 6.84	4.66, 6.07	0.33	T/C	2, 4	Glyma.18g281800		Homeodomain-like superfamily protein	
	ADZI			–12.96	9.22	5.23	0.33		1				
Gm20:32811819	DZI	20	32811819	42.23 ∼107.06	6.66 ∼8.69	12.78 ∼14.84	0.30 ∼0.31	C/T	1, 2, 6				
	GNI			59.81, 68.25	8.79, 10.44	11.59, 19.26	0.30 ∼0.31		1, 6				
	MDZI			93.33 ∼279.11	5.88 ∼8.00	6.04 ∼11.87	0.30 ∼0.31		1, 2, 3, 4, 6				
	MGNI			153.07 ∼204.05	9.47 ∼12.31	6.37 ∼21.93	0.30 ∼0.31		1, 2, 4, 6				
	ADZI			14.10 ∼26.37	5.75 ∼10.69	5.46 ∼21.18	0.30 ∼0.31		1, 2, 3, 6				
	TI			476.50 ∼866.84	7.86 ∼12.00	8.42 ∼21.35	0.30 ∼0.31		1, 2, 3, 4, 6				

DZE, daidzein; GLE, glycitein; GNE, Genistein; DZI, daidzin; GLI, glycitin; GNI, genistin; MDZI, malonyl daidzin; MGLI, malonyl glycitin; MGNI, malonyl genistin; ADGI, acetyl daidzin; AGLI, acetyl glycitin; AGNI, acetyl genistin; QTN, 1Quantitative trait nucleotide; Chr, chromosome; Effect, QTN effect for isoflavone content (μg g^–1^); R^2^, phenotype variation; MAF, minor allele frequency. Method 1–7 refers to mrMLM, FastmrMLM, FASTmrEMMA, pLAR-mEB, pKWmEB, and MLM, respectively.

### Dissection of stable quantitative trait nucleotides and candidate genes

**FIGURE 5:**
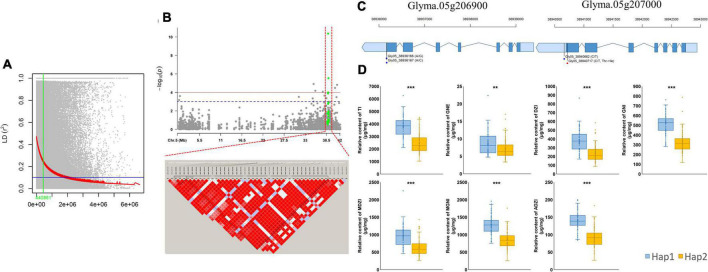
Linkage disequilibrium (LD) analysis of Gm05:38940662 (*GmGSTT1*) and variations in isoflavone contents between haplotypes. **(A)** Genome-wide average LD decay (R2) of MDP; **(B)** association mapping for Gm05:38940662 and LD analysis based on isoflavone contents; **(C)** gene models of *GmGSTT1* (Glyma.05g206900 and Glyma.05g207000); **(D)** isoflavone contents in different haplotypes.

To explore the genetic basis of isoflavone biosynthesis, we estimated the genome-wide average of LD decay using the genotype dataset of 25,646 SNPs. The LD half decay distance was approximately 441 kb (Figure 5A). Significant QTNs located around Gm05:38940662 (Glyma.05g207000) on chromosome 5 were detected by multiple models (MLM, mrMLM, FASTmrMLM, FASTmrEMMA, pLARmEB, pKWmEB, and ISIS EM-BLASSO) in 2020, and were related to the contents of six individual isoflavones (GNE, DZI, GNI, MDZI, MGNI, and ADZI) and TI. A total of 44 QTNs were detected in a 441-kb interval surrounding Gm05:38940662. Of these, four QTNs (Gm05:38936166, Gm05:38936167, Gm05:38940662 and Gm05:38940717) were significantly associated with variations in isoflavone contents (Figure 5B). Two of those QTNs (Gm05:38936166 and Gm05:38936167) were located at the 3’-untranslated region (UTR) of Glyma.05g206900 (a gene encoding glutathione S-transferase THETA1; *GSTT1*), and the other two (Gm05:38940662 and Gm05:38940717) were located at the 3’-UTR and caused non-synonymous substitutions in the coding sequence (CDS) of Glyma.05g207000 (a gene on chromosome 5 encoding another *GSTT1*). The QTNs at Gm05:38936166 and Gm05:38940662 caused transitions (A/G and C/T) and the QTN at Gm05:38936167 resulted in a transversion. The QTN at Gm05:38940717 resulted in a predicted amino acid substitution from threonine to isoleucine (Figure 5C).

To compare the isoflavone contents based on these four QTNs, a subset of 137 soybean mutants with variable isoflavone contents was divided into two haplotypes (Hap1 and Hap2) (Figure 5D). The mean contents of the following seven isoflavones were significantly different between Hap 1 (58 genotypes) and Hap 2 (79 genotypes): TI [hap1 (3806.44 μg g^–1^) and hap2 (2448.48 μg g^–1^), *p* < 0.001], GNE [hap1 (8.59 μg g^–1^) and hap2 (7.03 μg g^–1^), *p* < 0.01], DZI [hap1 (390.40 μg g^–1^) and hap2 (236.20 μg g^–1^), *p* < 0.001], GNI [hap1 (519.64 μg g^–1^) and hap2 (325.42 μg g^–1^), *p* < 0.001], MDZI [hap1 (974.80 μg g^–1^) and hap2 (632.11 μg g^–1^), *p* < 0.001], MGNI [hap1 (1281.23 μg g^–1^) and hap2 (857.82 μg g^–1^), *p* < 0.001], ADZI [hap1 (141.21 μg g^–1^) and hap2 (92.73 μg g^–1^), *p* < 0.001].

### Expression pattern the candidate gene *GmGSTT1* in a mutant

**FIGURE 6:**
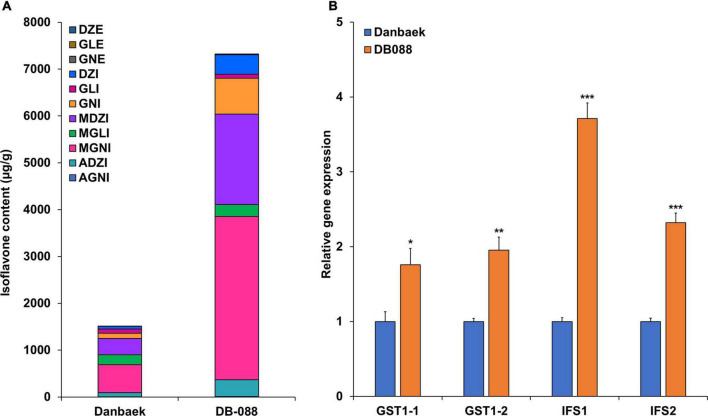
Transcript levels of *GmGSTT1* and other isoflavone biosynthetic genes in original cultivar (DB) and its mutant (DB-088), and isoflavone contents. **(A)** Isoflavone content in DB and DB-088; **(B)** relative gene transcript levels in DB and DB-088. All data are means ± SD (*n* = 5). The * *p* < 0.05, ** < 0.01, and ** < 0.001, Student’s *t*-test.

To explore the transcriptional patterns of *GmGSTT1* (Glyma.05g206900 and Glyma.05g207000) and those of the core isoflavone biosynthesis genes *IFS1* and *IFS2*, we conducted quantitative real-time PCR (qRT-PCR) analyses using seeds of Danbaek (DB) and its mutant (DB-088) with low and high isoflavone contents, respectively. These two lines were selected from the 189 members of the MDP as shown in Figure 6A. Consistent with the predicted effects of the QTNs, the contents of the isoflavones GNE, DZI, DZI, MDZI, MGNI, ADZI, and TI were significantly higher in seeds of DB088 than in those of DB. The qRT-PCR analyses confirmed differences in gene transcript levels between the two genotypes (Figure 6B). The relative transcript levels of *GST1-1* (Glyma.05g206900) and *GST1-2* (Glyma.05g207000) were 1.96-fold (*p* < 0.01) and 1.76-fold (*p* < 0.05) higher, respectively, in the seeds of DB088 than in the seeds of DB. Interestingly, *IFS1* and *IFS2* also showed higher transcript levels in the seeds of DB088 than in the seeds of DB, with similar transcriptional patterns to that of *GmGSTT1*. The transcript level of *IFS1* in seeds of DB-088 was 3.71-fold (*p* < 0.001) that in seeds of DB, and the transcript level of *IFS2* in DB-088 was 2.32-fold (*p* < 0.001) that in DB. These results suggested that *GmGSTT1* is involved in isoflavone synthesis in soybean seeds.

## Discussion

This core collection constructed by mutation breeding has previously been used in GWAS and linkage mapping studies to dissect important QTLs and candidate genes associated with quantitative and qualitative traits for crop improvement. Kim et al. (2019) constructed a bi-parental mapping population derived from the high-stearic acid mutant soybean Hfa180 with a three-base-pair deletion in exon 1 of *GmSACPD-C*. This mutation leads to a high stearic acid content and was developed using mutated alleles. In the population derived from this mutant, Kompetitive allele-specific PCR markers were used to identify a subset of high-stearic acid soybean lines. Hwang et al. (2020) performed GWAS analysis of a core rice mutant population (2,561 individuals) induced by gamma-ray irradiation. This population was subjected to abiotic stress treatments. Then, whole-genome resequencing analyses revealed a candidate gene for stress tolerance (Os02g0528900, encoding a PDR-like-ABC transporter) with two SNPs. Ma et al. (2022) used QTL mapping with bulk segregant analysis and GBS methods to detect the QTL for the dwarf phenotype of the mutant upland cotton line Ari1327. Thus, the use of GWAS or linkage mapping combined with a mutation breeding population is ideal for genome-wide mapping studies to explore the basis of complex agronomic traits.

To reveal causal genes associated with isoflavone content, we first conducted a GBS-based GWAS using multiple statistical methods, including MLM and mrMLM, to analyze data from a mutant mapping population consisting of 189 soybean mutant genotypes. The multi-locus random-SNP-effect mrMLM, which was developed by Zhang et al. (2020), includes six methods: mrMLM, FASTmrMLM, FASTmrEMMA, pLARmEB, pKWmEB, and ISIS EM-BLASSO. Unlike the conservative MLM model, the mrMLM method has been widely used to detect many important QTNs. The mrMLM estimated potentially significant QTNs based on empirical Bayes. Even if the significance threshold is less stringent, mrMLM have robust statistics, accuracy, short running time, and a low false positive rate (Cui et al., 2018; Guan et al., 2019; Zhang et al., 2020).

The isoflavone contents in the mutant population varied significantly between 2020 and 2021 because of complex heritability as well as external factors. In this study, the largest decrease was detected for aglycones (reduced by 50.39% in 2021 compared with that in 2020), followed by glucosides, total isoflavones, acetyl-glucosides, and malonyl-glucosides (reduced by 38.36, 24.28, 24.24, and 18.15% in 2021 compared with those in 2020, respectively). Thus, all of the isoflavones detected were affected by genotype, year, and the genotype × year interaction (Table 1). In previous studies, one soybean cultivar showed a 3.6-fold variation in isoflavone contents among different sites in the same year, and variations of up to 1.5-fold across different years (Eldridge and Kwolek, 1983); and the contents of individual isoflavones showed variations between 2 years ranging from 16.6% (MDZI) to 400% (GLE), and variations among eight planting locations ranging from 20.9% (MDZI) to 400% (GLE) (Hoeck et al., 2000). Factors that affect the contents of secondary metabolites in soybean seeds include temperature, location, sowing date, the type of fertilizer used, and the developmental stage of the seeds. Previous studies have demonstrated that high temperatures can lead to decreased contents of isoflavones and fatty acids (but not saponins) in soybean seeds. Similarly, isoflavone contents were found to be 5.8-fold lower in soybean varieties cultivated with early sowing dates (April and May) than in those cultivated with later sowing dates (June and July) (Tsukamoto et al., 1995). Another study showed that a shift from high to low temperature increased total isoflavone contents by 2.5-fold, with daidzein showing the largest increase of 3-fold (Lozovaya et al., 2005).

In our study, for those isoflavones showing a normal distribution in their contents in the MDP population, the broad-sense heritability ranged from 15% (GLE) to 86% (TI and ADZI) across the 2 years. Consistent with our results, a previous study detected heritability of TI ranging from 87.2 to 93.8% in individual and multiple environments. Meng et al. (2016) and Chu et al. (2017) detected broad-sense heritability of major isoflavone components ranging from 67.80% (GNE) to 83.80% (GLE) and from 87.2 to 93.8% (TI) across years and locations, respectively. However, Primomo et al. (2005) and Zeng et al. (2009) estimated the heritability for isoflavones to be lower than 60% based on multiple interactions. Overall, our results and those of other studies suggest that isoflavone contents in soybean seeds are more influenced by genetic effects than by year effects, indicating that isoflavone contents in soybean seeds are traits that can be genetically manipulated. We conducted correlation analyses for 12 types of isoflavones across two years (2020 and 2021), and detected strong positive correlations between total isoflavones and glucosides [DZI, GLI, and GNI (*r* = 0.64–0.94 in 2020 and *r* = 0.67–0.89 in 2021)]; and between total isoflavones and malonyl glucosides [MDZI, MGLI and MGNI (*r* = 0.64–0.94 in 2020 and *r* = 0.67–0.89 in 2021)]. We detected weaker positive correlations between total isoflavones and aglycones [DZE, GLE, and GNE (*r* = 0.46–0.82 in 2020 and *r* = 0.12–0.20 in 2021)]; and between total isoflavones and acetyl glucosides [ADZI and AGNI (*r* = 0.26–0.96 in 2020 and *r* = 0.47–0.95 in 2021)]. Across both years, DZI (*r* = 0.93***, *r* = 0.89***), GNI (*r* = 0.94***, *r* = 0.89***), MDZI (*r* = 0.94***, *r* = 0.94***), MGNI (*r* = 0.94***, *r* = 0.96***) and ADZI (*r* = 0.95***, *r* = 0.96***) showed significantly strong positive correlations with TI and with each other, consistent with the results of other studies (Azam et al., 2020; Yoon et al., 2021).

Previously, GWAS and QTL mapping have been used to detect candidate genes for quantitative and qualitative traits using natural populations and bi-parental populations, respectively. Recently, QTLs related to isoflavones have been detected by QTL mapping using a bi-parental population, an F_2_ population, and RILs. Cai et al. (2018) identified a novel QTL underlying isoflavone content using an RIL mapping population with 3469 bin markers based on restriction site-associated DNA tag sequencing. They detected 15 stable QTLs mapped onto ten chromosomes, among which qIF5-1 explained the largest proportion of variation in isoflavone content across environments (6.37–59.95%). In another study, specific-locus amplified fragment (SLAF) sequencing was used for high-density linkage mapping using 3,541 SLAF markers in multiple environments. That study detected 24 stable QTLs for isoflavone components, which explained 4.2–21.2% of the variations in their contents. They included four novel QTLs (qG8, qMD19, QMG18, and qTIF19) for GNI, MDZI, MGNE, and TI, respectively (Pei et al., 2018). A GWAS identified *GmMYB29*, a gene encoding a R2R3-type MTB transcription factor, which binds to the promoters of *IFS2* and *CHS8* to activate their expression. When *GmMYB29* was over-expressed or silenced by RNAi in soybean hairy roots, the isoflavone contents were increased by 1.6-fold to 3.3-fold or decreased by 2-fold, respectively (Chu et al., 2017). *GmMPK1*, encoding a mitogen-activated protein kinase, was detected in GWAS and linkage mapping studies using a bi-parental mapping population of soybean. This gene was found to be significantly associated with isoflavone contents and resistance to *Phytophthora* root rot (Wu et al., 2020). Another study detected *GmMYB176*, which encodes a transcription factor that binds to the *CHS8* promoter to regulate isoflavone biosynthesis. In further analyses, the isoflavone content decreased when *GmMYB176* was suppressed by RNAi, but it was not increased when *GmMYB176* was overexpressed (Yi et al., 2010). Overexpression of *GmMYB29* and *GmMPK1* led to increased isoflavone contents and enhanced tolerance to biotic and abiotic stresses, indicating that isoflavone biosynthesis is closely associated with resistance to external stress factors. Together, the results of those studies show that the expression of isoflavone biosynthetic genes, such as *PAL, 4CL, CHS8, CHR*, and *IFS2*, is activated by MYB, zinc, bZIP, and PLATZ transcription factors and their interactions in soybean.

In our study, we first screened for QTNs and candidate genes related to isoflavone contents using a mutant soybean population. and confirmed that isoflavone contents were significantly different (*p* < 0.001) between two haplotype groups. We identified QTNs in genes encoding transcription factors such as bZIP (Glyma.05g.223800), WRKY (Glyma.04g238300) and bHLH (Glyma.13g291400). We also detected a stable QTN (Gm05:38940662) on chromosome 5 in a gene encoding glutathione S-transferase THETA 1 (*GmGSTT1*), which was found to be associated with seven types of isoflavones (GNE, DZI, GNI, MDZI, MGNI, AZI, TI) in 2020 using multiple-GWAS models (Table 2). In the LD analysis based on Gm05:38940662, we detected 44 QTNs (Gm05:38513238 to Gm05:39363243) within a 441-kb surrounding Gm05:38940662. These four QTNs were located in the 3’-UTR and within non-synonymous coding regions in two *GmGSTT1* genes, and resulted in nucleotide changes (A/G, A/C, and C/T) resulting in an amino acid substitution in the first exon (Thr/Ile). We compared the contents of seven types of isoflavones between two haplotype groups: Hap1 with high isoflavone contents, and Hap2 with low isoflavone contents. Within these groups, the top 20% of Hap1 and bottom 20% of Hap2, the TI, GNE, DZI, MDZI, MGNI and ADZI of the 20% of Hap1 extremely increased up to 3.02-fold, 1.94-fold, 3.32-fold, 3.05-fold, 3.15-fold, 2.77-fold and 2.79-fold (*p* < 0.001) compared with the bottom 20% of Hap2, respectively. These data suggest that the four QTNs will be useful as informative molecular markers in breeding programs to increase the isoflavone content in soybean seeds.

Ionizing radiation induces mutations, including chromosomal alterations, SNPs, and insertions/deletions. Thus, it has been used extensively for crop improvement (Lee et al., 2014; Kim et al., 2019; Hwang et al., 2020; Ma et al., 2022). To confirm that the gene mutation detected in this study affected the transcript levels of *GmGSTT1*, we performed qRT-PCR analyses for DB-088, the mutant with the greatest increase in isoflavone content compared with the original cultivar DB (see Figure 6A). The genes *IFS1* and *IFS2* are known to be important determinants of isoflavone content, and were also up-regulated in DB-088 compared with those in DB. Previous studies have reported that non-synonymous SNPs in exons of *IFS1* and *IFS2* not only greatly reduced isoflavone contents in soybean seeds, but also affected responses to biotic and abiotic stresses (Cheng et al., 2008, 2010). In the present study, the expression profile of *GmGSTT1* was similar to that of two *IFS* genes, suggesting that *GmGSTT1* is also related to variations in isoflavone content. The four QTNs in *GmGSTT1* were related to increased contents of seven types of isoflavones (up to 1.65-fold) (Figure 5D).

The NCBI database lists 133 soybean *GST* genes. Members of this family are well characterized detoxification enzymes that are involved in stress tolerance and nodulation in soybean roots. They are a ubiquitous class of enzymes that participate in the antioxidant system, thereby decreasing the accumulation of reactive oxygen species (Dalton et al., 2009; Dixon et al., 2010). Overexpression of *GsGST* genes has been shown to increase tolerance to abiotic stresses such as salt, drought, cold and heavy metals, and biotic stresses such as insect attack and virus infection (Takesawa et al., 2002; Ji et al., 2010). Members of the GST family also play roles in the accumulation of secondary metabolites such as anthocyanins and cinnamic acid. Some GSTs have been reported to contribute to the formation of other natural products such as the sulfur-containing phytochemicals in Brassicaceae species (Dixon et al., 2010; Czerniawski and Bednarek, 2018). In maize, the anthocyanin biosynthetic gene Bronze-2 is involved the accumulation of anthocyanins in the vacuole. During this process, anthocyanins conjugate with GST, and the anthocyanin-GSH complex is transported into the vacuole *via* a glutathione pump (Marrs et al., 1995). Activation of GST catalyzes the binding of GSH to ABC transporters, enabling ATP hydrolysis to direct translocation across the membrane. ATP-binding cassette (ABC) transporters are well known for role in sequestration of flavonoids glycosides, glucuronides and glutathione conjugates into the vacuole (Petrussa et al., 2013). As mentioned above, members of the GST family are involved in many aspects of growth and development during the whole life span of plants. Isoflavones are precursors of phytoalexins, and their accumulation is induced in response to hormones and external stimuli such as insect attack. Like anthocyanins, isoflavones accumulate in the vacuole. Thus, it is possible that *GmGSTT1* conjugates with isoflavones for transport and storage, like it does with anthocyanins.

This is the first GWAS analysis associated with isoflavone content using a mutant soybean population with multiple GWAS-models across two years. We have identified many significant QTNs, and detected four stable QTNs in the gene *GmGSTT1*. Our findings provide evidence that *GmGSTT1* affects isoflavone contents in soybean seeds. The results of this study include genetic information that will be useful for crop improvement, specifically for the production of soybean lines with stable and high isoflavone contents.

## Data availability statement

The datasets presented in this study can be found in online repositories. The names of the repository/repositories and accession number(s) can be found in the article/Supplementary material.

## Author contributions

S-JK, B-KH, and J-WA conceived and revised the manuscript. JMK designed the experiment and drafted the manuscript. JMK, YJL, SHE, JSS, and NNH phenotyped the isoflavone content. JIL and D-GK genotyped the GBS data. JMK, JIL, and D-GK analyzed the GWAS data. All authors contributed to the article and approved final manuscript.

## Footnotes

1
https://mvd.iaea.org

2
https://cran.r-project.org/web/packages/mrMLM/index.html

3
https://phytozome-next.jgi.doe.gov/

4
https://soybase.org/
